# The associations between fasting blood glucose levels and mortality of SFTS in patients

**DOI:** 10.1186/s12879-021-06463-3

**Published:** 2021-08-05

**Authors:** Yin Zhang, Yu Zhang, Yuanhong Xu, Ying Huang

**Affiliations:** Department of Clinical Laboratory, The First Affiliated Hospital of Anhui Medical University, No. 218 Jixi Rd, Hefei, Anhui Province China

**Keywords:** SFTSV, Cohort study, Fasting blood glucose, Outcomes, Mortality

## Abstract

**Objective:**

To identify the correlation between the level of at-admission fasting blood glucose (FBG) with poor outcomes in hospitalized patients suffering from severe fever with thrombocytopenia syndrome (SFTS).

**Methods:**

Between April 1 and December 1, 2020, the list of hospitalized patients affected with SFTS infection was provided by the Infectious Disease Department at First Affiliated Hospital of Anhui Medical University, followed by the collection of information I.e., gender, age, diabetic history and the level of FBG on admission.

**Results:**

In this study, a total of 77 patients were included and were categorized into three groups (< 5.6, 5.6–6.9, and ≥ 7.0 mmol/l) on the basis of their glucose level in the blood. The obtained results revealed that among three groups considerable variations were observed in leukocytes, FBG, D-Dimer, aspartate aminotransferase (AST), tumor necrosis factor-α (TNF-α), fibrin degradation products (FDP), and interleukin (IL)-10 level. Correlation analysis indicated a linear negative correlation between PLT and FBG (*r* = − 0.28, *P* = 0.01), however, a linear positive correlation was observed between AST, IL10, D-Dimer, and FDP levels and FBG (*P-*value < 0.05). Multivariate statistical analysis results shown that there was significant difference between group comparison (F = 17.01, *P* < 0.001) and interaction between group and time (F = 8.48, *P* < 0.05); but there was no significant difference between time point comparison (F = 0.04, *P* = 0.96). With the prolongation of time, the changes of FBG were different between survivor group and non-survivor group. The FBG in survival group shown a downward trend; The non-survivor group shown an upward trend.

**Conclusions:**

Elevated level of FBG has been correlated with hypercoagulability, inflammation, and lower PLT in SFTS patients. The measurement of FBG level can help in evaluating the inflammatory process, hypercoagulability, and prognosis of patients suffering from SFTS. FBG can predict the prognosis of SFTS. It is necessary to pay attention to the role of FBG in the process of treatment in patients with SFTS.

## Introduction

Severe fever with thrombocytopenia syndrome (SFTS) is an infectious disease. For the first time, the underlined infection was reported in China (2011) and then the cases were appeared in South Korea and Japan [1–3]. SFTS has been caused by SFTS bunyavirus (SFTSV) and its transmission occurs through tick bites while sometimes, it can be transmitted through aerosol and blood transfer [4, 5]. Humans of all ages can be infected by SFTS but the people of old ages i.e.*, 50* ≥ are at higher risk due to compromised immune systems [6]. The patients infected with SFTS may be asymptomatic or symptomatic with a common febrile illness that can lead to hypovolemic shock and failure of multiple organs [7]. A mortality rate of 50% has been reported for SFTS [8].

The earlier studies have revealed that the mortality rate in SFTS has a positive correlation with diabetes. However, the association of FBG with the severity and fatality rate of the disease has not been revealed. In this view, it is important to identify whether FBG can be used as a prognostic marker in SFTS for early detection of severe cases and reduce the death rate.

In the current study, the clinical data of 77 patients in First Affiliated Hospital of Anhui Medical University were evaluated to identify the FBG predictive value in the severity and fatality in SFTS.

## Materials and methods

### Study design and the collection of data

In the current study, the clinical data of patients (affected with SFTS) was collected from the first affiliated hospital of Anhui Medical University between 1st April 2020 and 1st December 2020. Eighty-one patients (suffering from SFTS) were admitted into the hospital in which 4 patients were ruled out for the underlined reasons: (1) no FBG data available at admission (2) having previously diagnosed diabetes. All patients were classified into three groups on the basis of WHO guidelines in terms of FBG at admission (< 5.6, 5.6–6.9, and ≥ 7.0 mmol/l).

### Ethical approval

This study conformed to the Declaration of Helsinki and was approved by the Medical Ethics Committee of the First Affiliated Hospital of Anhui Medical University. In this retrospective study, informed consent was waived by using anonymous clinical data.

### Laboratory examination

Regular blood tests were carried out in all patients, including blood routine test, coagulation profile measurement, CRE (creatinine), FBG, ALT (alanine aminotransferase), AST (aspartate aminotransferase), CK (creatine kinase), and LDH (lactate dehydrogenase) levels. Inflammatory markers i.e.*,* PCT (procalcitonin), CRP (C-reactive protein), TNF-α (tumor necrosis factor-α), and interleukins (IL-1β, IL-2R, IL-6, IL-8, and IL-10) were also determined in all patients. RT-PCR was employed for the detection of SFTSV.

### Statistical analysis

The statistical analysis was conducted via SPSS (version 24.0) and Graphpad Prism (version 9.0). In descriptive statistics, the median (IQR) was for continuous variables, and counts and proportions were for categorical variables. Chi-square or Mann-Whitney U test was used for evaluating variations in parametric and non-parametric demographic data in SFTS survivors and non-survivors. For continuous variables, the Kruskal Wallis test was conducted to compare three categories (FPG < 5.6, 5.6–6.9, and ≥ 7.0 mmol/l) while Fisher’s exact or chi-square test was carried out for categorical variables. The correlation between the level of FBG and variables was evaluated by Spearman’s bivariate simple correlation analysis. General linear models were used to compare FBG of different times among patients with SFTS between survivor and non-survivor groups. Results were regarded to be statistically considerable at a two-tailed *P* value of less than 0.05.

## Results

SFTSV RNA tests were carried out in which a total of 77 patients were found to be SFTS positive. In SFTS positive patient’s there were 35 males and 42 females. 16 of 77 cases died and their median duration from hospital admission to death was 4.5 days. 61 of 77 cases were discharged and their median duration from hospital admission to discharge was 12 days. Herein, our study reveals a significant comparison between the clinical characteristics of deceased and survivor patients.

The treatment regimens are shown in the Table 1. Patients in the non-survival group, compared with patients in survival group, were more likely to receive the treatment of oxygen therapy (nasal cannulation, mask oxygenation, high-flow nasal cannula oxygen therapy, non-invasive positive pressure ventilation or invasive mechanical ventilation) (49.1% vs. 100%) and blood product therapy (47.5% vs. 56.2%). The 2 groups showed no significant difference in corticosteroid treatment, ribavirin, antibacterial treatment, antifungal treatment, or intravenous immunoglobulin (Table 1).

**Table 1:** The clinical characteristics between survivors and non-survivors

	Survivors *N* = 61	Non-survivors *N* = 16	*P* values
Age, years, median (IQR)	63 (55–69)	66 (61–72)	0.26
Gender (Male/Female)	34/27	8/8	0.682
Time from onset to admission (days)	5 (4–7)	5 (5–7)	0.969
Duration of hospital admission (days)	12 (9–16)	4.5 (1.2–7.7)	< 0.001
Treatment, n (%)			
Oxygen therapy	30 (49.1)	16 (100)	< 0.001
Ribavirin	59 (96.7)	16 (100)	0.463
Antibacterial treatment	34 (55.7)	11 (68.7)	0.347
Antifungal treatment	14 (22.9)	1 (6.2)	0.133
Glucocorticoids	26 (42.6)	10 (62.5)	0.156
Intravenous immunoglobulin	52 (85.2)	16 (100)	0.102
Use of blood product	29 (47.5)	9 (56.2)	< 0.001
Fasting blood glucose (mmol/L)	6.9 (5.3–9.5)	10.1 (12.9)	0.015
Leukocytes (×10^9^/L)	2.32 (1.6–3.9)	2.3 (1.4–3.5)	0.551
Neutrophils (×10^9^/L)	1.3 (0.8–2.3)	1.2 (0.8–2.3)	0.90
Platelet (×10^9^/L)	46 (34–68)	40 (25–52)	0.189
Procalcitonin (ng/ml)	0.11 (0.05–0.22)	0.54 (0.15–0.89)	0.001
C-reactive protein (mg/L)	3.1 (0.8–9.2)	4.7 (2.0–12.8)	0.177
Aspartate aminotransferase (U/L)	120 (89.5–232)	457 (128–706)	0.005
Alanine aminotransferase (U/L)	75 (43.5–92)	97 (59–178)	0.034
Creatinine (μmol/L)	67.5 (58–88.1)	94.2 (76–166)	0.001
lactate dehydrogenase (U/L)	827 (562–1556)	1720 (1008–5761)	0.002
Creatine kinase (U/L)	316 (116–731)	580 (185–3563)	0.096
PT, secs.	13.5 (12.8–13.9)	13.6 (12.8–15.2)	0.397
APTT, secs.	50.4 (42.8–54.8)	67.7 (54.4–85.5)	< 0.001
D-Dimer (μg/mL)	1.87 (1.27–3.61)	6.7 (2.8–15.7)	< 0.001
Fibrin degradation product (μg/mL)	5.98 (3.77–12.3)	19.7 (8.9–63.2)	0.001
Serum amyalse (U/L)	99 (30–681)	99.5 (55–392)	0.56
Serum lipase (U/L)	559 (27–2000)	581 (105–1576)	0.95
Interleukin-1β (pg/ml)	8.1 (5.0–13.5)	22.7 (10.9–36.0)	< 0.001
Interleukin-2R (U/ml)	1106 (841–1381)	1714 (1267–2803)	0.001
Interleukin-6 (pg/ml)	10.8 (5.0–16.3)	68.7 (20.4–140.0)	< 0.001
Interleukin-8 (pg/ml)	20.5 (14.9–32.4)	73.5 (34.4–232.5)	0.001
Interleukin-10 (pg/ml)	36 (10.4–63.6)	117 (53.8–275.5)	< 0.001
Tumor necrosis factor-α (pg/ml)	18.4 (14.8–24.0)	47.4 (27.3–116.3)	< 0.001

According to the obtained data, the survivors were comparatively younger than those non-survivors patients. Non-survivors had relatively elevated FBG levels i.e.*,* 10.1 vs. 6.9, *P* = 0.015, and shorter hospital stay (4.5 vs 12.0, *P* < 0.001) as compared to the survivor’s patients. Based on their biochemical considerations, non-survivors were found to be with elevated level of AST (457 vs 120 U/L, *P* = 0.005), ALT (97 vs 75 U/L, *P* = 0.034), CRE (94.2 vs 67.5 umol/L, *P* = 0.001) and LDH (1720 vs 827 U/L, *P* = 0.002) levels. Based on the inflammatory biomarkers, the non-survivors had significantly higher PCT (0.54 vs 0.11 ng/ml, *P* = 0.001), IL-1β (8.1 vs 22.7 pg / ml, *P* < 0.001), IL-2R (1714 vs 1106 U/ml, *P* = 0.001), IL-6 (68.7 vs 10.8 pg / ml, *P* < 0.001), IL-8 (73.5 vs 20.5 pg/ml, P = 0.001), IL-10 (117 vs 36 pg / ml, *P* < 0.001) and TNF-α (47.4 vs 18.4 pg/ml, *P* < 0.001, Table 1).

Based on the FBG levels, the selected population was further categorized into three classes. The percent of deaths were 5.3, 22.2, and 27.5% in groups A, B, and C, respectively, as presented in Table 2. Among these three groups; FBG, AST, D-Dimer, FDP, IL-10, TNF-α levels, and leukocyte counts were found to be considerably varied (all *P* < 0.05). As compared with those in group A, leukocyte counts were found to be considerably lower, while AST, D-Dimer, FDP, IL-10, and TNF-α concentration were recorded comparatively higher (all *P* < 0.05).

**Table 2:** Comparison of laboratory parameters and baseline characteristics among the three groups of SFTS patients

Variables	Group A (*n* = 19)	Group B (*n* = 18)	Group C (*n* = 40)	*p*-Value
Age, years, median (IQR)	62.0 (51.0–68.0)	58.5 (53.8–70.0)	66.0 (63.0–70.0)	0.059
Sex				
Female, n (%)	8 (42.1)	8 (44.4)	26 (65.0)	0.158
Male, n (%)	11 (57.9)	10 (55.6)	14 (35.0)	
Onset symptoms				
Fever, n (%)	19 (100)	17 (94.4)	39 (97.5)	0.568
Cough, n (%)	6 (31.6)	8 (44.4)	18 (45.0)	0.144
Expectoration, n (%)	3 (15.8)	5 (27.8)	16 (40.0)	0.162
Muscular soreness, n (%)	11 (57.9)	12 (66.7)	16 (40.0)	0.131
Fatigue, n (%)	15 (78.9)	11 (61.1)	32 (80.0)	0.278
Diarrhoea, n (%)	7 (36.8)	10 (55.6)	20 (50.0)	0.491
Time from onset to admission, median (IQR), d	7.0 (5.0–8.0)	5.0 (4.0–7.0)	5.0 (5.0–7.0)	0.357
FBG (mmol/L)	4.91 (4.42–5.22)	6.16 (5.96–6.54)	10.23 (8.71–12.93)	< 0.01
Leukocytes (×10^9^/L)	4.02 (1.63–5.07)	2.80 (1.93–3.34)	1.94 (1.30–2.93)	0.026
Neutrophils (× 10^9^/L)	2.06 (0.87–2.73)	1.38 (0.94–2.07)	1.12 (0.73–1.98)	0.159
Platelet (×10^9^/L)	54.0 (34.0–69.0)	45.0 (40.7–73.0)	40.5 (30.0–59.0)	0.108
CRP (mg/L)	3.9 (0.8–10.43)	3.57 (0.69–6.57)	3.1 (1.15–10.17)	0.635
Procalcitonin (mg/L)	0.11 (0.05–0.45)	0.09 (0.06–0.15)	0.20 (0.11–0.37)	0.067
IL-1β (pg/mL)	6.85 (5.0–11.95)	9.02 (5.0–18.8)	9.74 (5.0–16.6)	0.489
IL-2R (U/mL)	955 (748–1353)	1151 (941–1369)	1352 (1031–1678)	0.106
IL-6 (pg/mL)	7.58 (3.2–24.0)	12.8 (6.63–59.9)	13.0 (7.14–34.3)	0.172
IL-8 (pg/mL)	17.3 (12.8–28.3)	25.5 (17.05–50.5)	27.2 (17.7–47.9)	0.057
IL-10 (pg/mL)	11.65 (7.2–27.3)	24.9 (11.0–56.9)	61.7 (43.2–139.0)	0.0001
TNF-α (pg/mL)	18.2 (13.3–27.0)	16.6 (14.2–25.5)	23.2 (17.0–43.0)	0.042
AST (U/L)	110 (84.0–147)	125.5 (98.0–204.7)	227.5 (101.7–425.5)	0.021
ALT (U/L)	78.0 (39.0–89.0)	69.5 (44.5–98.5)	86.5 (54.7–120.5)	0.422
CRE (μmol/L)	72.8 (53.6–86.2)	83.9 (59.5–101.8)	71.5 (60.1–91.3)	0.592
LDH (U/L)	723 (531–1314)	798 (500–1353)	1177 (754–2322)	0.059
CK (U/L)	239 (93–686)	362 (107–1679)	406 (158–1007)	0.427
PT (S)	13.7 (12.7–14.5)	13.3 (12.8–13.6)	13.5 (12.7–14.2)	0.477
APTT (S)	45.7 (39.9–58.9)	51.2 (48.7–54.1)	54.5 (47.5–65.3)	0.184
FIB (g/L)	2.98 (2.6–3.85)	2.77 (2.42–3.25)	2.78 (2.35–3.46)	0.429
D-Dimer (μg/ml)	1.54 (1.24–1.89)	2.47 (1.44–3.34)	3.84 (1.75–8.93)	0.003
FDP (μg/ml)	4.92 (3.55–5.78)	6.28 (4.03–11.31)	11.79 (6.01–30.58)	0.002
Serum amyalse (U/L)	81 (30–353)	88.5 (34–458)	109 (43–681)	0.264
Serum lipase (U/L)	518 (44–2000)	559 (58–2000)	625 (27–2000)	0.416
Secondary infection, n (%)	8 (42.1)	5 (27.8)	22 (55.0)	0.148
Median length of in-hospital stay (days), median (IQR)	10.0 (9.0–12.0)	10.5 (6.5–15.0)	11.0 (8.0–16.0)	0.465
Mortality (n, %)	5.3%	22.2%	27.5%	0.14

**Fig. 1:**
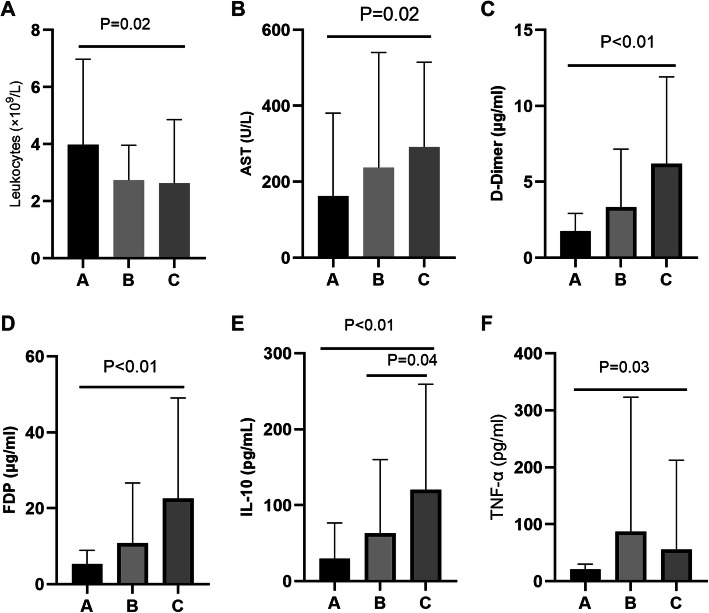
Inflammatory factors in blood and FBG among the three groups

**Fig. 2:**
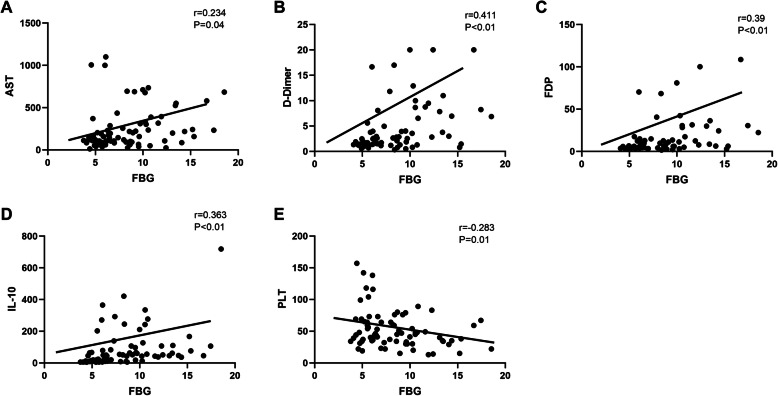
Correlation analysis of inflammatory factors, platelet, and FBG level

According to the pairwise comparison, the difference between groups B and C was statistically significant in terms of IL-10 levels, as shown in Fig. 1. Spearman’s bivariate simple correlation analysis revealed that the concentration of FBG has a positive correlation with AST (*r* = 0.234, *P* = 0.04), D-Dimer (*r* = 0.411, *P* < 0.01), FDP (*r* = 0.39, *P* < 0.01) and IL-10 (*r* = 0.363, *P* < 0.01) levels. Moreover, the levels of FBG were found to be negatively correlated with PLT levels (*r* = − 0.283, *P* = 0.01) as shown in Fig. 2.

**Fig. 3:**
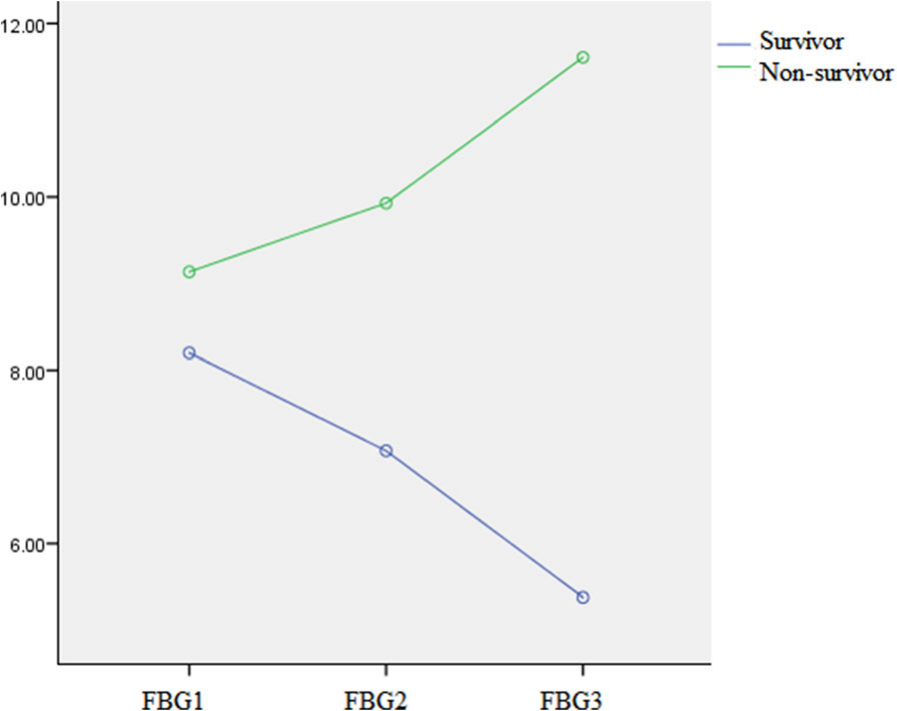
Changes of fasting blood glucose over time

We collected serial FBG and used general linear models to compare FBG of different times among patients with SFTS between survivor and non-survivor groups. Multivariate statistical analysis results show that there was significant difference between group comparison (F = 17.01, *P* < 0.001) and interaction between group and time (F = 8.48, *P* < 0.05); but there was no significant difference between time point comparison (F = 0.04, *P* = 0.96). With the prolongation of time, the changes of FBG were different between survivor group and non-survivor group. As shown in Fig. 3, the FBG in survival group shown a downward trend; The non-survivor group shown an upward trend.

## Discussion

In hospitalized patients, the admission glucose level is considerably associated with outcomes, and a high level of glucose predicts elevated lengths of stay, failure of organs, and increased mortality. For instance, hyperglycemia predicts raised mortality and the development of organ dysfunction in patients with sepsis [9]. The elevated level of glucose may evaluate prehospitalization comorbidity, including diabetes or side effects of drugs i.e.*,* corticosteroids, or may characterize acute stress reactions correlated with severe illness. Elevated glucose level results in an osmotic diuresis with hypovolemia and loss of electrolyte and can attenuate immune responses. Hyperglycemia activates the formation of advanced glycation end products which interacts with the lipids, proteins, and cell membrane. Interaction with the cellular membrane can result in endothelial dysfunction. Infection may cause stress and stimulation of the sympathetic nervous system. The pancreas can also be attacked by the SFTS virus. Due to these factors the patients infected with SFTS might be more susceptible to hyperglycemia. In this view, the level of glucose pattern in patients was analyzed to evaluate the associated risk factor of hyperglycemia.

Herein, we evaluated the influence of FBG level on the prognosis of non-diabetic patients, associated with SFTS. Survivors showed a reduced level of FBG when compared with non-survivors. Besides, survivors had a low level of proinflammatory cytokines than non-survivors, which suggests that cytokine storm and sustained inflammatory effect may be linked with the severity of SFTS. According to the recently reported data, SFTS positive patients had an elevated concentration of G-CSF, MCP1, IP10, IL1-RA, IL6, and IL10 [10, 11]. It has been reported that hyperglycemia is correlated with considerably increasing oxidative stress, and level of inflammatory cytokines, and drastically altering the balance between inflammatory and anti-inflammatory cytokines [12]. Acute hyperglycemic condition is associated with the alteration of innate immune responses, which may up to some extent demonstrate the poor outcomes in those SFTS positive individuals who developed hyperglycemia [13]. This study also analyzed that patients with an elevated level of FBG had a comparatively high concentration of inflammatory cytokines such as TNF-α and IL-10. Moreover, in comparison with the other two groups, patients in the high FBG group were found to be with low leukocyte counts. The underlined results revealed that increase FBG is correlated with immunity and infection in SFTS positive and non-diabetic patients. One of the possible reason of infection development is stress-induced hyperglycemia because the elevated concentration of glucose negatively affect the essential components of the innate immune system [14], and can induce aberrant glycosylation of proteins, enzymes and immunoglobulins, followed altering the adaptive immune system [15]. High glucose concentration decreases the migration and phagocytosis ability of neutrophils [13].

In this study, out of 77 SFTS positive patients, 59 patients were found to be with the pancreatic injury. These patients were found to be comparatively more vulnerable to diarrhea and anorexia. In the 59 patients, pancreatic enzymes elevations were mostly mild, and no patients had clinically severe pancreatitis. Meanwhile, 47 of the 59 patients had blood glucose abnormalities. None of the patients demonstrated radiographic evidence of pancreatitis. The concentration of amylase and lipase were in the range of 34 to 681 U/L, and 301 to 2000 U/L, respectively. According to these results, the primary target of the virus is maybe the pancreas, followed by causing series injury, leading to a decreased level of insulin production. The interaction of SFTS with the pancreatic endocrine system complicates the situation for SFTS positive patients. On the other hand, most patients took antipyretics before admission, which could cause drug-related pancreatic injury. In addition, due to the kidneys malfunction, amylase and lipase can not be completely removed, resulting in a transient increase in pancreatic enzymes. So far, there is no precise definition of pancreatic involvement during SFTSV infection. Further work is needed in this topic to better explain the real impact of SFTSV in pancreatic damage and its role in human pathogenesis.

Previously reported studies demonstrated that some viruses can directly invade on beta cells of the pancreas [16]. Similarly, several latest studies reveal that the viral replicating process takes place in the mammalian pancreas, followed by eliciting inflammation-associated responses but does not cause diabetes [17]. Mechanical shock can also result in hypoxic pancreatic damage [18]. This supports the evidence that pancreatic injury is higher among patients with more severe disease. We do not believe that the possible relate between SFTS infection and pancreatic function should be ruled out although we were incapable of identifying diabetogenic activity for the SFTS virus.

In the current study, serum samples of SFTS positive patients revealed an elevated level of aspartate and alanine aminotransferases, creatine kinase, creatinine, D-dimer, lactate dehydrogenase, fibrin degradation products, and activated partial-thromboplastin time (APTT). These aberrantly altered parameters are indicative of pathological lesions in the heart, kidney, liver, and coagulation systems of SFTS positive patients. In SFTS, hepatocytes were found to be not infected with SFTSV, while several SFTSV-NP antigen-positive cells were detected in the liver [19, 20]. In this view, it is demonstrated that in SFTS positive patients, the liver damage might be due to a subordinate pathological event rather than due to direct destruction caused by SFTSV.

As with other viral hemorrhagic fevers, critical features of SFTS evident in applicable animal models include damage to secondary lymphoid organs, widespread coagulopathy, and induction of a ‘cytokine storm’ that may lead to septic shock [21]. Besides, elevated permeability of endothelium results in endothelial damaging that leads to death. Endothelial damage triggers the intrinsic cascade of coagulation and also activates the aggregation of platelet adhesion and degranulation which imbalance homeostasis. Finally, the underlined conditions result in disseminated intravascular coagulation (DIC) and diffuse hemorrhage because of elevated consumption level of coagulation factors. In SFTS, the liver function has been impaired due to the cytopathic effect of viruses which results in decreasing the formation of plasma coagulation factors, as the underlined factors are mostly formed in the liver.

The present study has some limitations. Firstly, this is a retrospective study of a single-center dataset while the sample size is also small. Therefore, further conformation is necessary along with a multi-centered study, larger sample size, and patients of multi-ethnic origins. Secondly, the multivariate regression analysis might not be conducted to evaluate whether the elevated FBG level was an independent risk factor for the mortality in patients affected with SFTS due to many deaths. Thirdly, whether the elevated FBG level in patients (affected with SFTS) is transient is not clear due to the short time of observation and needs further evaluation. Eventually, our sample size limited the glycemic target research for SFTS patients with hyperglycemia.

In summary, at-admission hyperglycemia is a bad prognostic indicator and needs proper detection and cure and this postulate has been supported by our data. The measurement of FBG level can help in evaluating the inflammatory process, hypercoagulability, and prognosis of patients suffering from SFTS. In the present study, significantly controlling glucose level would result in a good prognosis in patients (affected with SFTS). So, Glycemic testing has been suggested for all patients (affected with SFTS) because mostly SFTS patients are susceptible to glucose metabolic disorders. Therefore, it is necessary to pay attention to the role of FBG in the process of treatment in patients with SFTS.

## Data Availability

The data that support the findings of this study are available from the corresponding author upon reasonable request.
